# Worldwide Research Trends on Artemisinin: A Bibliometric Analysis From 2000 to 2021

**DOI:** 10.3389/fmed.2022.868087

**Published:** 2022-05-06

**Authors:** Yankai Dong, Lina Liu, Jie Han, Lianqing Zhang, Yi Wang, Juan Li, Yuexiang Li, He Liu, Kun Zhou, Luyao Li, Xin Wang, Xue Shen, Meiling Zhang, Bo Zhang, Xiaofei Hu

**Affiliations:** ^1^College of Life Sciences, Northwest University, Xi'an, China; ^2^General Medical Department, Nankai Hospital, Tianjin, China; ^3^Department of Radiology, Southwest Hospital, Third Military Medical University (Army Medical University), Chongqing, China; ^4^Clinical Laboratory, Ankang Hospital of Traditional Chinese Medicine, Ankang, China

**Keywords:** bibliometrics, artemisinin, VOSviewer, network, hotspot

## Abstract

**Objective:**

Artemisinin is an organic compound that comes from Artemisia annua. Artemisinin treatment is the most important and effective method for treating malaria. Bibliometric analysis was carried out to identify the global research trends, hot spots, scientific frontiers, and output characteristics of artemisinin from 2000 to 2021.

**Methods:**

Publications and their recorded information from 2000 to 2021 were retrieved through the Web of Science Core Collection (WoSCC). Using VOSviewer and Citespace, the hotspots and trends of studies on artemisinin were visualized.

**Results:**

A total of 8,466 publications were retrieved, and for the past 22 years, the annual number of publications associated with artemisinin kept increasing. The United States published most papers. The H-index and number of citations of the United States ranked first. The University of Oxford and MALARIA JOURNAL were the most productive affiliation and journal, respectively. A paper written by E.A. Ashley in 2011 achieved the highest global citation score. Keywords, such as “malaria,” “artesunate,” “plasmodium-falciparum,” “*in-vitro*,” “artemisinin resistance,” “plasmodium falciparum,” “resistance,” and “artemether-lumefantrine,” appeared most frequently. The research on artemisinin includes clinical research and animal and cell experiments.

**Conclusion:**

The biosynthesis, drug resistance mechanism, and combination of artemisinin have become more popular than before. Studies on artemisinin treating coronavirus disease 2019 (COVID-19) have been carried out, and good research results have been obtained.

## Introduction

Artemisinin was first extracted from Artemisia annua by Chinese researchers in 1972, and it has a good therapeutic effect on malaria (1, 2). At present, the World Health Organization (WHO) believes that artemisinin-based combination therapy (ACT) is still the most effective method to treat malaria (3). Due to the great effect of artemisinin in treating malaria, researchers have set off an upsurge of artemisinin research. The research on artemisinin is not limited to antimalarial effect; it also focuses on its efficacy as antivirus, antitumor, anti-inflammatory, and antiparasitic (except malaria) (4, 5). Many artemisinin derivatives have been developed to improve the oral bioavailability, short half-life, and low solubility of artemisinin (6–9). However, artemisinin-resistant strains of *Plasmodium* have appeared in some parts of Southeast Asia in recent years. During 3-day ACTs, the clinical features of patients suffering from malaria are the slow clearance rate of *Plasmodium* and *Plasmodium* could not be eliminated completely (10). Due to the long history of application and the rapid development of modern science and technology, studies on artemisinin need a comprehensive summary and generalization to determine the research trend. New technologies and concepts are also being introduced. Thus, the trends and hotspots of artemisinin research have changed in recent years, bringing challenges to researchers. Groups of professors and scholars have made great efforts and published many papers thus far. However, a summing-up commentary is lacking. A comprehensive review of this field is essential, and old and new participants in this field could benefit from it. As an interdisciplinary science, bibliometrics could analyze all knowledge carriers quantitatively by using statistical and mathematical methods (11, 12). As a convenient method, bibliometrics could estimate development trends and reveal key research directions by analyzing database and publication features. In addition, it could provide effective evidence to guide experimental strategies and funding decisions (13, 14).

During these years, fruits of bibliometric analysis, such as aristolochic (15), osteomyelitis (16), knee revision (11), tuberculosis (17), butyrophilins (18), and macrophages associated with acute lung injury (13), have been reported. However, no bibliometric study on artemisinin could be found. In this study, bibliometric analysis and a comprehensive review of artemisinin research were performed to investigate the trends of this research and provide suggestions for future studies.

## Methods

### Data Sources and Search Strategies

**Figure 1:**
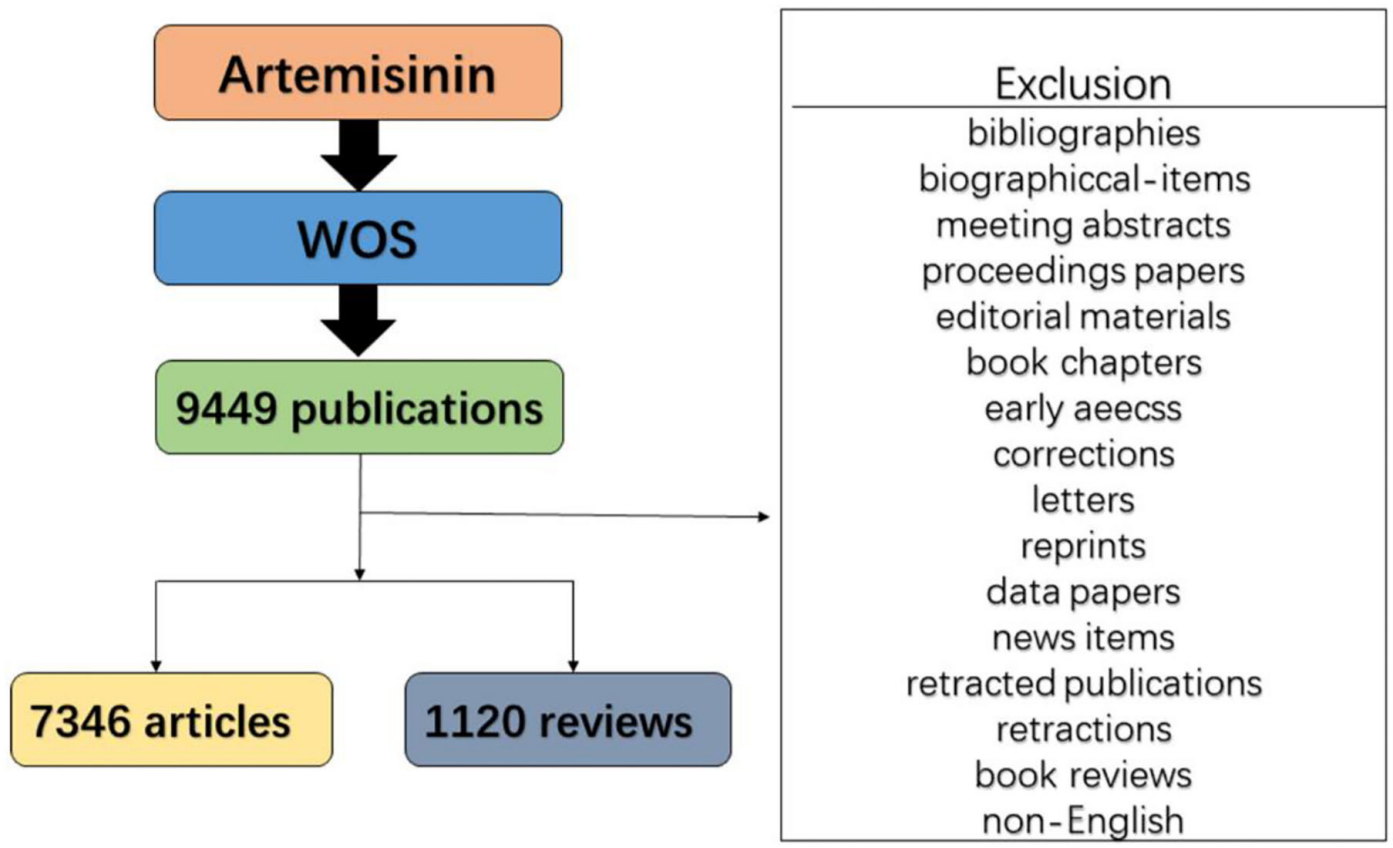
Flowchart of the screening process.

Due to a standardized and comprehensive dataset for export and wide use in academia, the Web of Science Core Collection (WoSCC) was used to compile the publication dataset in this study. The timespan of the retrieval was set between 2000 and 2021 to explore the global research trends in artemisinin study for a long time. The retrieval was carried out on 18 January 2022. At the initial stage of bibliometric analysis, “artemisinin” was used as the search term, and 9,449 publications were retrieved from the WoSCC database. Several queries were successively carried out as a part of retrieval. For example, “arteannuin” or “artemisinine” or “artemisine” or “artemisinin” were used as the search term. Finally, “artemisinin” was used as the retrieval term because it led to almost every relevant search result. In different types of relevant publications (i.e., meeting abstracts, editorial materials, preceding papers, letters, news items, corrections, book chapters, early access, book reviews, data papers, reprints, bibliographies, and biographical items), only articles and reviews written in English were included in the following analysis. A total of 7,346 articles and 1,120 reviews were retrieved and analyzed. Figure 1 shows the retrieval strategy in this research.

### Bibliometric Analysis

On the basis of WoSCC, text data were obtained, and further analysis was carried out with VOSviewer (version 1.6.10) and Citespace (version 5.7.R5). In accordance with the data, bibliometric indicators were extracted, including the number of publications (NPs) and the number of citations (NC) without self-citations. In 2005, Hirsch proposed the H-index (19), which introduced scientific measurement into a new context from the method and concept. According to its original definition, a researcher would have an H-index if he or she published H papers, and each of the papers at least had been cited H times. This simple, novel, and special method combines the NC with the number of papers, comprehensively considering the quantity and quality of scholars' papers, and breaks the phenomenon of paying too much attention to local citations in the past. As the most important breakthrough in scientometrics indicators since the 21st century, in the following 10 years, the research on H-index could become one of the most important hotspots in the field of scientometrics and academic evaluation (19, 20). A network of co-occurrence keywords, in which the research hotspots associated with artemisinin could be illustrated clearly, was also constructed, and the bursts of keywords and references are often used to detect new research trends in the field (21).

## Results

### Overview of Research on Artemisinin

In accordance with the retrieval strategy, 8,466 articles and reviews published from 2000 to 2021 were retrieved, of which the total NC was 149,185 and the average NC per publication was 29.33. The H-index for all publications was 174.

### Annual Trends in NP

**Figure 2:**
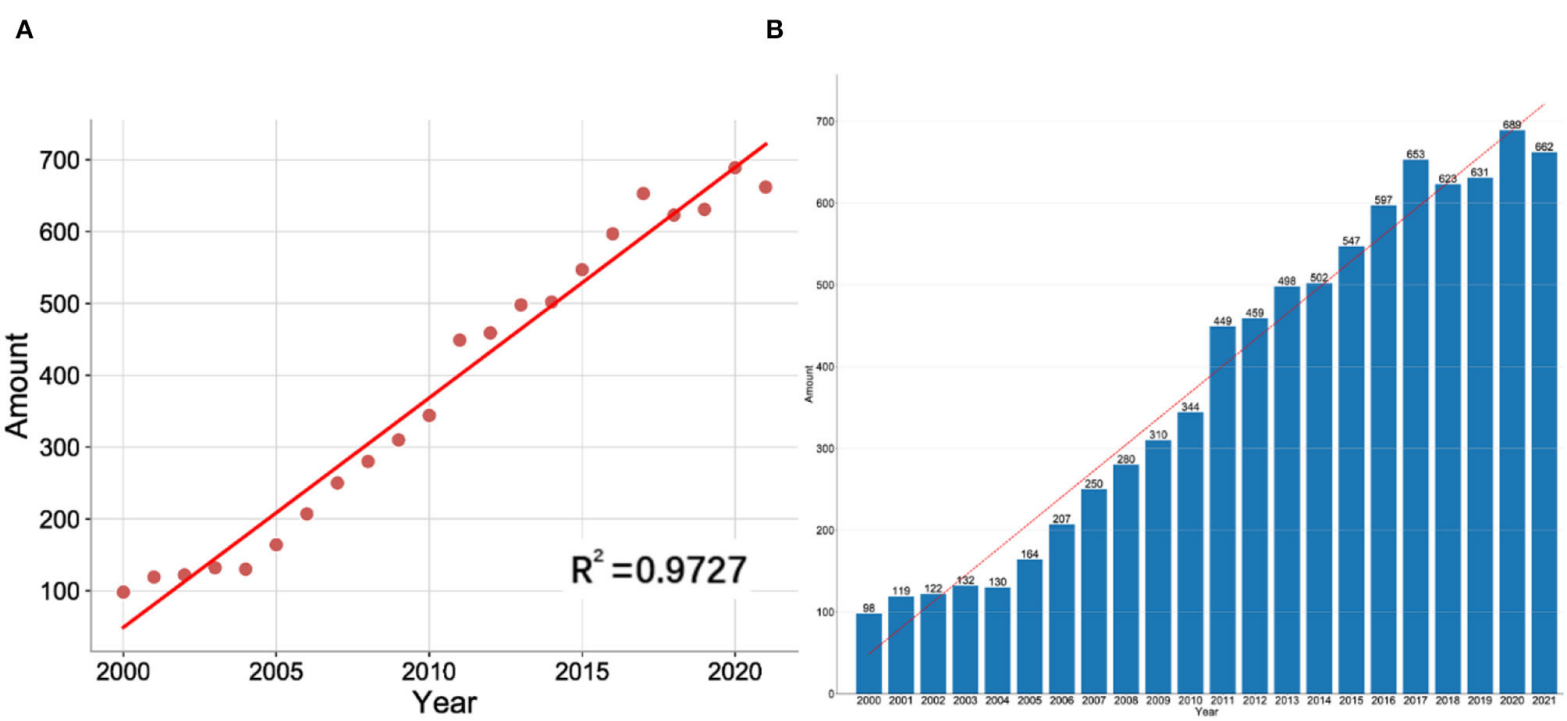
**(A)** Curve fitting of the total annual growth trend of publications (*R*^2^ = 0.9727). **(B)** The number of publications by year over the past 22 years.

Figure 2A shows the polynomial fitting curve of the annual trend of publication. The annual NP was obviously related to publication year, and the correlation coefficient *R*^2^ reached 0.9727. Figure 2B shows the annual NP associated with artemisinin. In general, despite the fluctuation over the past 22 years, the annual NP rose from 98 in 2000 to 662 in 2021, reaching its peak in 2020.

### Characteristics of Countries

**Figure 3:**
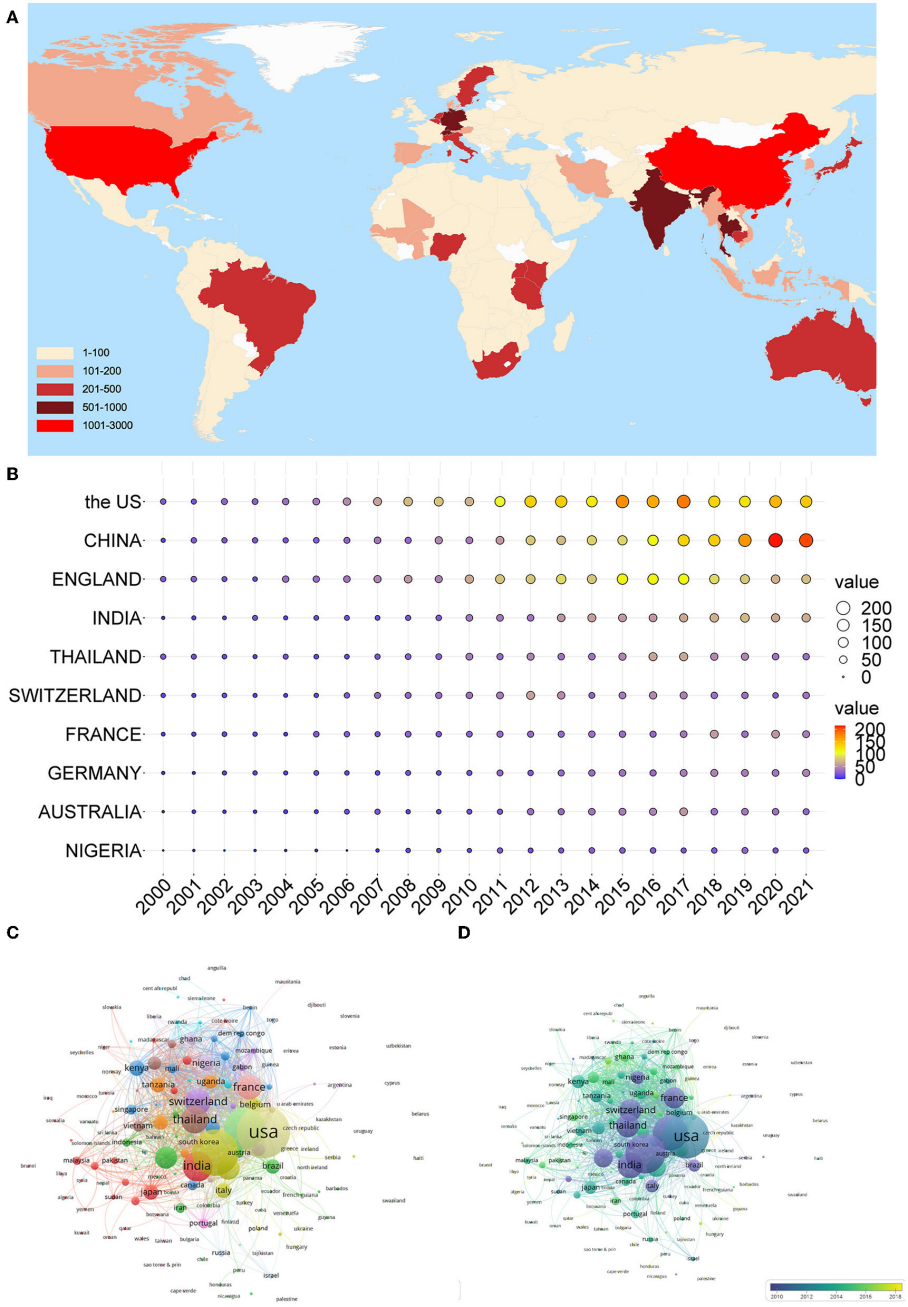
Countries in artemisinin research. **(A)** Geographical distribution of global output; **(B)** annual output trend of the top 10 productive countries; **(C)** visual cluster analysis of cooperation among countries; and **(D)** timeline visualization of cooperation among countries.

Analysis of the publications in different countries could reflect the importance that a country attaches to this field and its influence in this field to some extent. From 2000 to 2021, 157 countries published research articles on artemisinin (Figure 3A). The top 10 output countries/regions according to the NP are listed in Figure 3B. Since 2000, the NP in the United States and China has maintained steady growth. Fluctuations could be observed in the NP from England, with the largest NP being 114 in 2015. In general, these findings showed that the research on artemisinin has entered a stage of rapid development and attracted extensive attention. As shown in Table 1, the United States had the most publications (2,050), followed by China (1,641) and England (1,347). Papers from the United States were cited 76,901 times, accounting for 51.55% of the total citations. England (56,951) and Thailand (31,993) ranked second and third, respectively. Besides, the United States achieved the highest H-index (131), more than two times the number for India (54) and Nigeria (31). England had a moderately lower NP but higher H-index, NC, and average per item than China. Thailand achieved the highest average per item (53.74), followed by England (49.06), indicating that the publications of the two countries were of high quality. The average per item in China, India, and Nigeria was relatively low, suggesting that the quality of publications in these countries needs to be improved. As shown in the visualized international collaboration network, close cooperation was found between countries (Figure 3C). India, France, Thailand, and Switzerland carried out research in this area earlier than other countries (Figure 3D).

**Table 1:** Publications in the top 10 productive countries/regions.

**Rank**	**Country**	**NP**	**NC**	**H-index**	**Average per item**
1	The United States	2,050	76,901	131	44.3
2	China	1,641	31608	87	24.04
3	England	1,347	56,951	116	49.06
4	India	797	14,254	54	20.16
5	Thailand	687	31,993	91	53.74
6	Switzerland	634	25,565	81	43.7
7	France	591	19,363	72	35.94
8	Germany	510	18,253	74	39.2
9	Australia	497	17,922	71	39.06
10	Nigeria	279	5,822	31	22.42

### Performance of Affiliations and Authors

**Figure 4:**
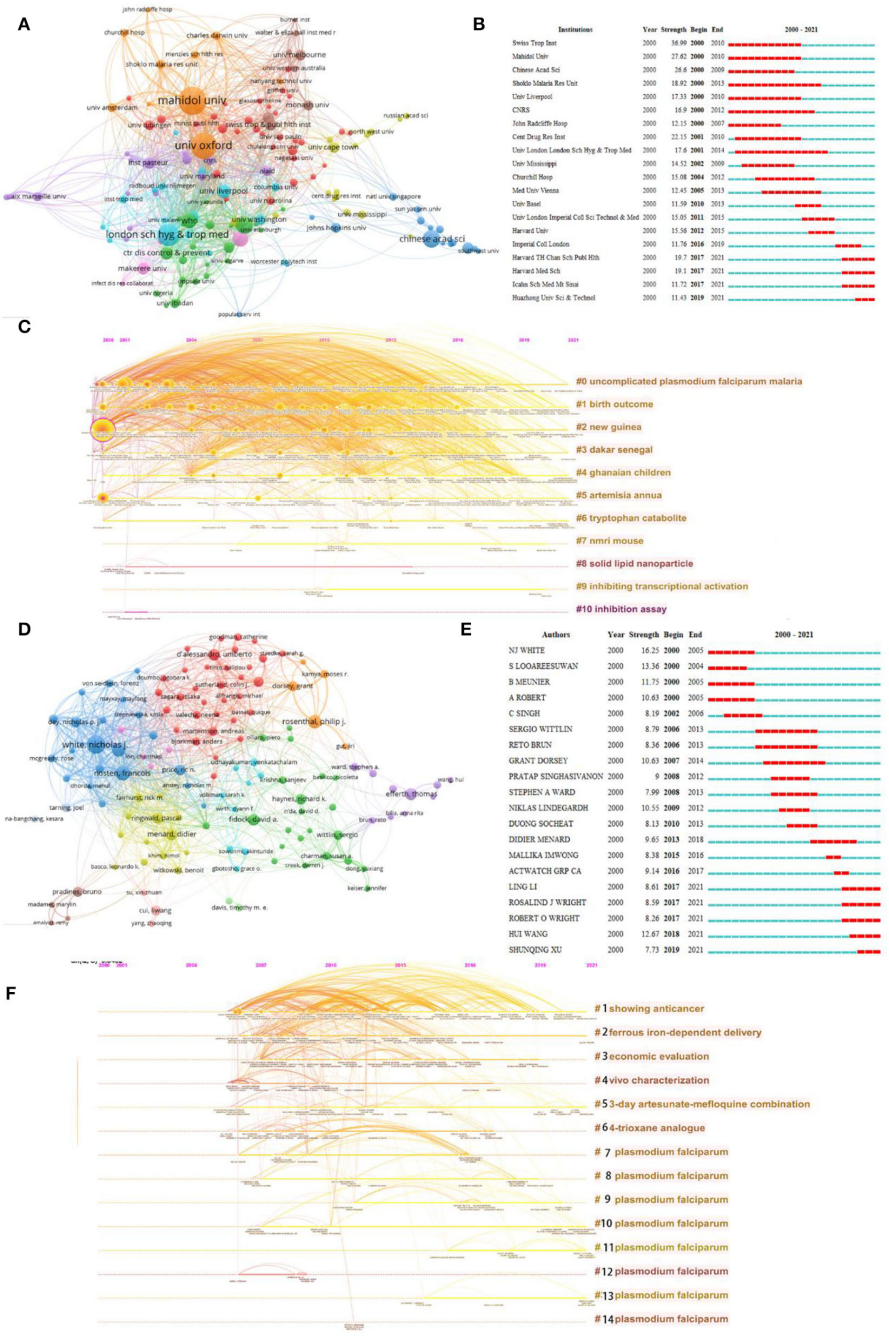
Visualization of active affiliations and author analysis. **(A)** Analysis of cooperation among affiliations. **(B)** Top 20 representative burst affiliations. **(C)** Timeline distribution of cluster analysis of affiliation. **(D)** Analysis of cooperation among authors. **(E)** Top 20 representative burst authors. **(F)** Timeline distribution of cluster analysis of the author.

The top 10 affiliations with the most publications associated with artemisinin are presented in Table 2. The University of Oxford had the highest NP (524), NC (30,311), and H-index (90), followed by Mahidol University and the University of London. In addition, the University of Oxford achieved the highest average per item (64.68), followed by Mahidol Oxford Tropical Medicine Research Unit (62.05) and Mahidol University (61.05). Moreover, 30% of the top 10 affiliations were from England. Mahidol University and the University of Oxford took up the core position (Figure 4A). Figure 4B shows the 20 most representative affiliations in terms of burst strength, burst duration, and burst time. Swiss Trop Instant had the highest burst strength. Eleven clusters were identified (Figure 4C), which included “uncomplicated *Plasmodium falciparum* malaria,” “birth outcome,” and “new guinea.” Table 3 shows the top 10 authors with the most publications. They published 988 papers, accounting for 11.67% of the total NP. Their NC was 76,490, accounting for 51.27% of the total NC. White, NJ from the Mahidol University in Thailand ranked first in the research field of artemisinin, followed by Nosten, F from the University of Oxford in England and Dondorp, AM from Mahidol University in Thailand. As shown in Table 3, Dondorp, AM had the highest average per item (108.36). Day, NPJ had the lowest number of documents. However, Day, NPJ ranked third in terms of average per item (98.03), showing that the quality of the author's publications was high. In addition, 30% of the top 10 authors were from Thailand. The co-occurrence network of authors is shown in Figure 4D. Among all authors, NJ White had the highest burst strength (Figure 4E). The cluster analysis of the authors showed 14 clusters, including “showing anticancer,” “ferrous iron-dependent delivery,” and “economic evaluation” (Figure 4F).

**Table 2:** The top 10 productive affiliations.

**Rank**	**Affiliations**	**Country**	**NP**	**NC**	**H-index**	**Average per item**
1	University of Oxford	England	524	30,311	90	64.68
2	Mahidol University	Thailand	520	28,283	86	61.05
3	University of London	England	514	20,756	76	44.05
4	London School of Hygiene and Tropical Medicine	England	460	18,661	72	44.45
5	University of California System	The United States	304	16,816	62	58.02
6	Mahidol Oxford Tropical Medicine Research Unit	Thailand	284	15,691	62	62.05
7	Center national de la recherche scientifique	France	274	9,932	50	39.01
8	World Health Organization	Switzerland	268	13,528	57	53.22
9	Chinese Academy of Sciences	China	256	7,393	50	31.04
10	University of Basel	Switzerland	224	9,044	49	42.68

**Table 3:** The top 10 authors with the most publications.

**Rank**	**Author**	**Country**	**Affiliations**	**NP**	**NC**	**H-index**	**Average per item**
1	White, NJ.	Thailand	Mahidol Univ	204	18,468	70	96.29
2	Nosten, F.	England	Univ Oxford	138	13,024	53	98.79
3	Dondorp, AM.	Thailand	Mahidol Univ	107	11,022	44	108.36
4	Rosenthal, PJ.	The United States	Univ Calif San Francisco	91	4,546	39	53.79
5	D'alessandro, U.	Belgium	Inst Trop Med Prince Leopold	79	2,645	28	35.2
6	Efferth, T.	Germany	Johannes Gutenberg Univ Mainz	79	5,026	44	71.95
7	Price, RN.	England	John Radcliffe Hosp	78	3,684	32	49.6
8	Fidock, DA.	The US	Columbia Univ	71	6,535	38	97.52
9	Menard, D.	France	Inst Pasteur	71	4,884	31	72.85
10	Day, NPJ.	Thailand	Mahidol Univ	70	6,656	34	98.03

### Performance of Journals and Analysis of Co-citation

**Figure 5:**
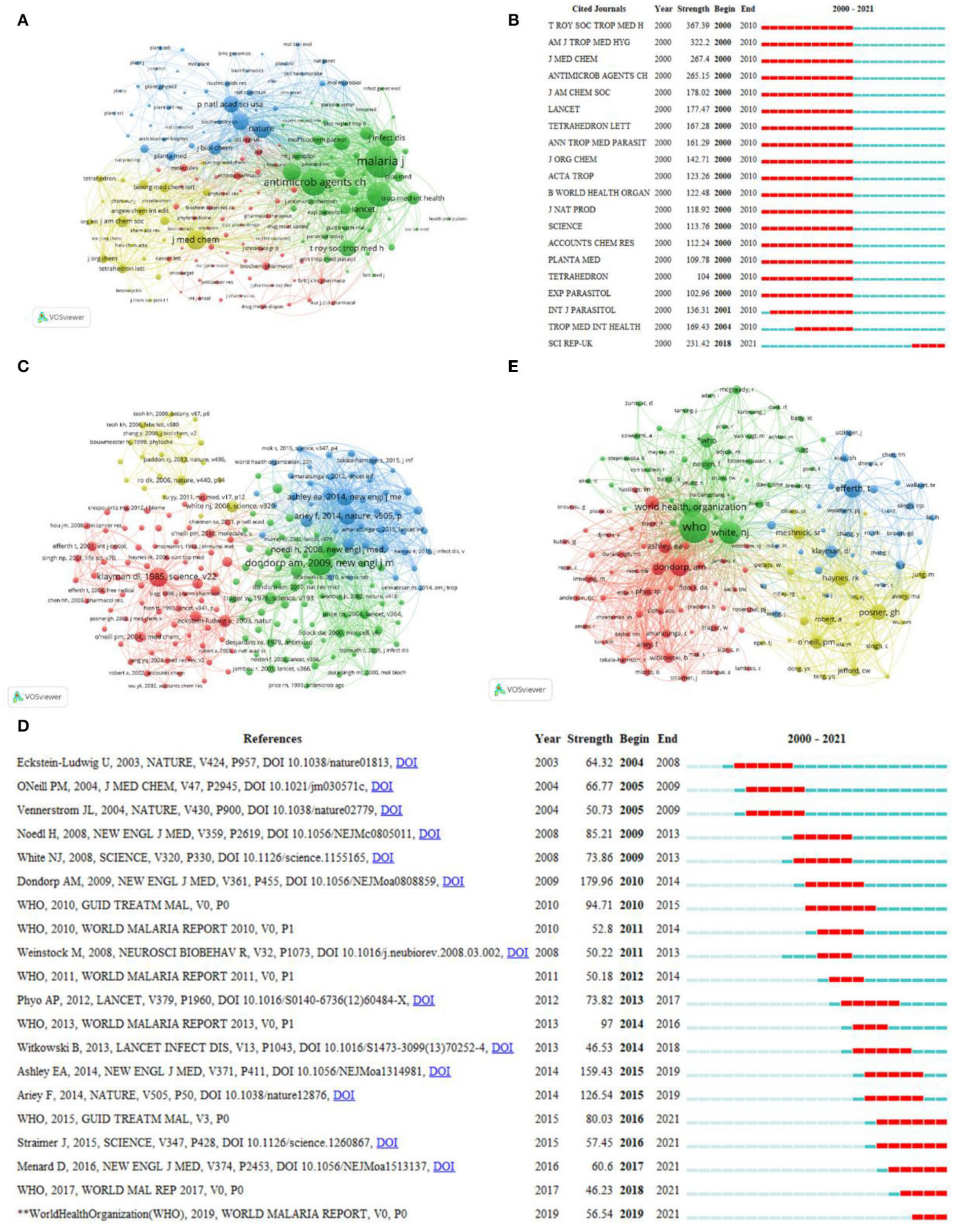
Visualization of the cited journal, co-cited reference, and co-cited author analysis. **(A)** Co-occurrence network of cited journals. **(B)** Top 20 representative burst cited journals. **(C)** Co-occurrence network of co-cited reference. **(D)** Top 20 representative burst co-cited references. **(E)** Co-occurrence network of the co-cited author.

As presented in Table 4, MALARIA JOURNAL (1,021 publications, IF: 2.979) published the greatest number of papers related to artemisinin. ANTIMICROBIAL AGENTS AND CHEMOTHERAPY (337 publications, IF: 5.191) and PLOS ONE (231 publications, IF: 3.24) ranked second and third, respectively. The top 10 journals published approximately 30% of the papers (2,390/28.23%). Of these top 10 journals, except for ANTIMICROBIAL AGENTS AND CHEMOTHERAPY (IF: 5.191) and JOURNAL OF MEDICINAL CHEMISTRY (IF: 7.446), the rest were journals with low IF (defined as lower than 5.000), indicating that researchers should improve the quality of their papers and conduct more in-depth and valuable research. MALARIA JOURNAL notably had the highest H-index and NC, and JOURNAL OF MEDICINAL CHEMISTRY exhibited the highest average per item. Although BIOORGANIC MEDICINAL CHEMISTRY had the lowest NP, its H-index was higher than that of BIOORGANIC MEDICINAL CHEMISTRY LETTERS, SCIENTIFIC REPORTS, ACTA TROPICA, and MOLECULES. The co-occurrence network of the cited journals is shown in Figure 5A. The top three journals with the highest citations were MALARIA JOURNAL, ANTIMICROBIAL AGENTS AND CHEMOTHERAPY, and PLOS ONE. When two articles appear in the references of the third citation together, a co-citation relationship is formed (22). Figure 5B shows the 20 most representative journals in terms of burst strength, burst duration, and burst time. In the co-citation network, the line between two nodes implies that two papers were cited in one publication. The size of nodes represented the total number of co-citations of a document. Besides, nodes were divided into different clusters by using different colors. In view of the great number of cited references, 100 was set as the minimum NC of a reference. Among the 174,484 references cited by the retrieved publications, 206 references were selected for co-citation analysis (Figure 5C). The top 10 co-cited references are shown in Supplementary Table 1. The co-citation of the paper written by Dondorp in 2009 was cited 1,360 times and in the first place, followed by the paper written by Klayman in 1985 and by Ashley in 2014. A total of 75 references were included in cluster 1 (in red), which mainly concentrated on the research on the synthesis, mechanism, efficacy, and pharmacokinetics of artemisinin. Cluster 2 (in green) was mainly about the study of antimalarial methods and drug resistance. Cluster 3 (in blue) paid attention to resistance in *Plasmodium falciparum* malaria and countermeasures. The main content of cluster 4 (in yellow) focused on the biosynthesis, extraction, and separation of artemisinin. Among all co-cited references, the paper written by Eckstein-Ludwig in 2003 had the highest burst strength (Figure 5D). The minimum number of co-citations of an author was set as 193 due to the large amounts of authors. Among the 104,510 authors cited by retrieved publications, 158 co-cited authors were selected for analysis (Figure 5E). The top 10 co-cited authors are shown in Supplementary Table 2. White, NJ had the most citations (2,924), followed by WHO (2,233) and Efferth, T (2,103). In terms of total link strength, White, NJ ranked first (45,050), followed by Posner, GH (33,037), and Haynes, RK (30,363). Although Posner, GH had a slightly low citation, the author's total link strength (33,037) was higher than that of WHO, Efferth T, and Dondorp AM.

**Table 4:** The top 10 most active journals.

**Rank**	**Journal**	**NP**	**NC**	**IF(2020)**	**H-index**	**Average per item**
1	Malaria Journal	1,021	19,072	2.979	64	21.7
2	Antimicrobial Agents And Chemotherapy	337	10,875	5.191	56	34.35
3	PLOS ONE	231	7,036	3.24	47	31
4	American Journal Of Tropical Medicine And Hygiene	227	6,022	2.345	41	27.41
5	Journal Of Medicinal Chemistry	124	6,119	7.446	49	51.52
6	Bioorganic Medicinal Chemistry Letters	111	2,482	2.823	29	23.3
7	Scientific Reports	88	1,414	4.38	21	16.26
8	Acta Tropica	86	2,557	3.11	26	30.26
9	Molecules	84	1,952	4.412	21	23.63
10	Bioorganic Medicinal Chemistry	81	2,215	3.641	31	27.81

### Analysis of Global Citation Score

**Figure 6:**
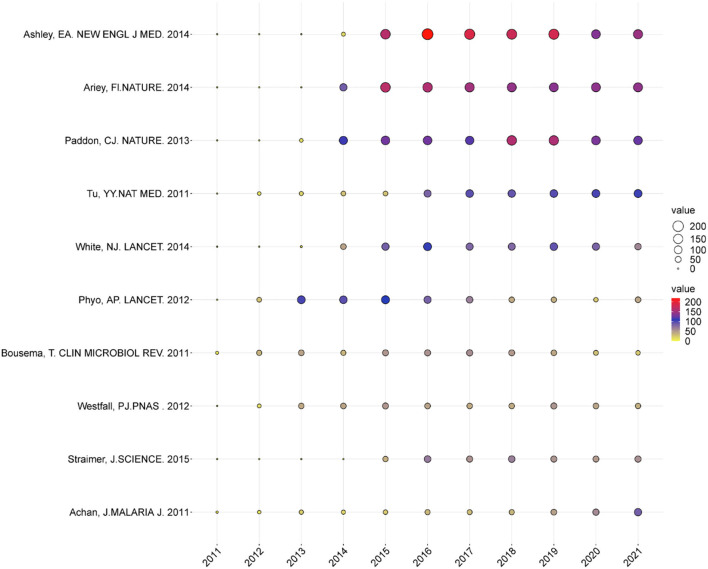
The yearly number of global citations of papers with high global citations (GCS). The size and colors of the circle represent the GCS of papers.

Figure 6 shows the annual number of global citation scores (GCS) of the top 10 publications. The paper written by Ashley in 2014 had the highest GCS (1,231). In this study, E.A. Ashley concluded that the prevalent resistance of *Plasmodium falciparum* to artemisinin in Southeast Asia was related to kelch13 mutations. Extended courses of ACTs were effective in areas where the standard 3-day treatment has failed (23). Ariey et al. linked artemisinin resistance to mutations in the PF3D7_1343700 Kelch propeller domain (“K13-Propper”) through whole-genome sequencing of the artemisinin-resistant parasite in Cambodia and parasite strains in Africa. Kelch13 (K13) propeller allele mutation was concentrated in Cambodia, and resistance was widespread. As a useful molecular marker, K13 polymorphism was conducive to controlling artemisinin resistance and avoiding its global spread (24). Paddon et al. proved the complete biosynthetic pathway of artemisinin. In addition, they developed a chemical process that could convert artemisinic acid to artemisinin with a chemical source of singlet oxygen (25). Tu described the discovery and application of artemisinin (2). White et al. reviewed the clinical, pathological, and epidemiological features of malaria and the means of prevention and treatment; they pointed out that a high supply of ACT is an effective means to reduce the incidence rate and mortality of malaria (26). Phyo et al. found that parasite clearance half-lives greatly lengthened and identified 148 multi-locus parasite genotypes (27). Bousema et al. summarized the epidemiology and infectivity of *Plasmodium falciparum* and *Plasmodium vivax* gametocytes in relation to malaria control and elimination (28). Westfall et al. described the progress toward the goal of developing semisynthetic artemisinin by fermenting the artemisinin precursor from engineered Saccharomyces cerevisiae and chemically converting it to dihydroartemisinin acid, which could then be converted to artemisinin (29). Straimer et al. provided solid evidence to identify and eliminate artemisinin-resistant malaria parasites (30). Achan et al. reviewed the historical uses of quinine, and its current use was considered. ACT offered a better choice than quinine, although in resource-limited settings, maintaining a stable supply of ACT was difficult (31). In any case, the focuses of artemisinin research in these publications were different, but they were all pioneering, which had a good guiding significance for the follow-up research in this field.

### Analysis of Keywords

**Figure 7:**
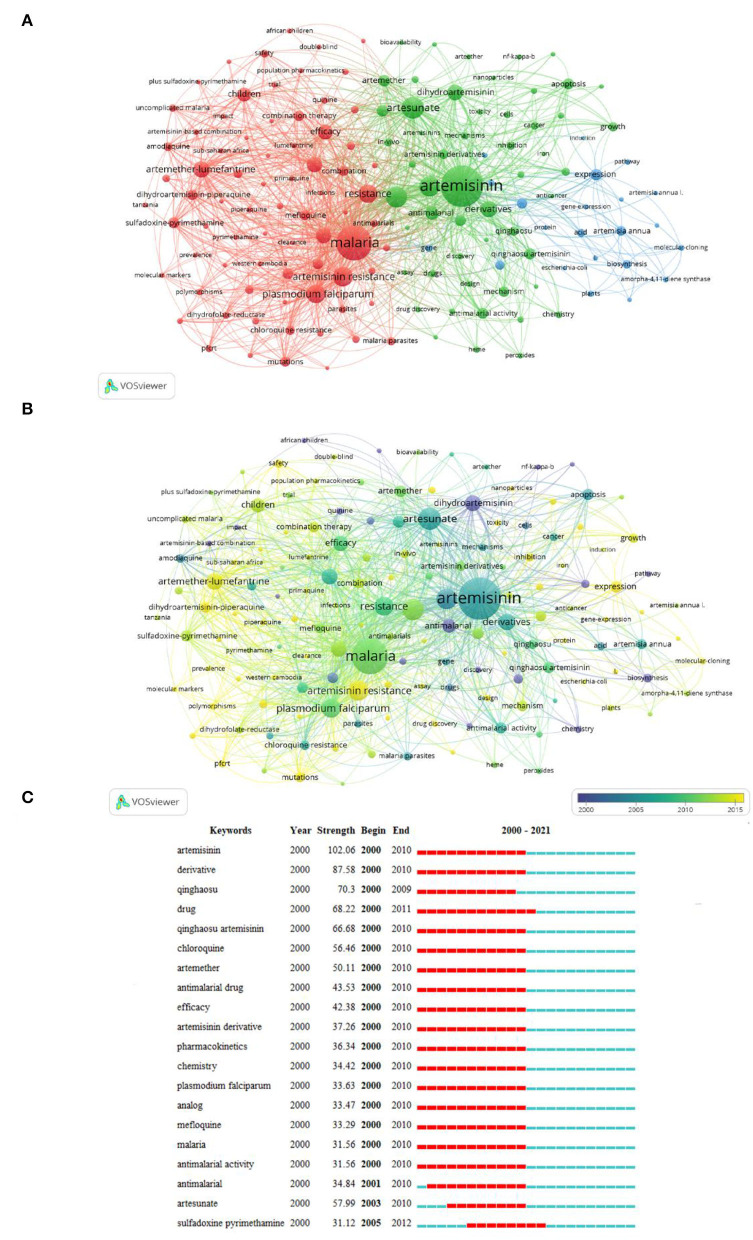
Network on keywords of artemisinin. **(A)** The 150 keywords that occurred more than 80 times were divided into 3 clusters by different colors: cluster 1: red, cluster 2: green, cluster 3: blue. The size of the nodes represents the frequency of occurrences. **(B)** Visualization of keywords according to the average publication year (APY). Keywords in yellow appeared later than that in blue. **(C)** Top 20 representative burst keywords.

The keywords extracted from abstracts and titles of the 8,466 publications were analyzed (Figure 7). As shown in Figure 7A, cluster 1 majorly focused on clinical research of artemisinin on malaria treatment, especially for children in Africa, including efficacy and drug resistance. Cluster 2 mainly reflected on basic research and explored the main mechanism of artemisinin in the treatment of diseases. Cluster 3 focused on the biosynthesis mechanism of artemisinin. The top 20 keywords in frequency are shown in Supplementary Table 3. The most frequent keywords were “artemisinin,” “malaria,” “artesunate,” “plasmodium-falciparum,” “*in-vitro*,” “artemisinin resistance,” “plasmodium falciparum,” “resistance,” and “artemether-lumefantrine,” indicating that studies associated with artemisinin mainly involved basic research, clinical research, and structure modification. As shown in Figure 7B, in accordance with the average publication year (APY), all keywords were divided into different types of colors through VOSviewer. Compared with artemisinin, artesunate, and other earlier research keywords, nanoparticle (2017.37), piperaquine (2016.51), artemisinin biosynthesis (2016.88), and drug discovery (2016.11) have become major topics in this field. Comparison between Figures 7A,B showed that artemisinin combination and biosynthesis were the research hotspots in recent years. Figure 7C shows the 20 most representative keywords in terms of burst strength, burst duration, and burst time. The findings showed that in the early stage of artemisinin research, the chemical synthesis of artemisinin and its derivatives, antimalaria, and combination drugs are the research hotspots.

### Bibliographic Coupling Analysis

**Figure 8:**
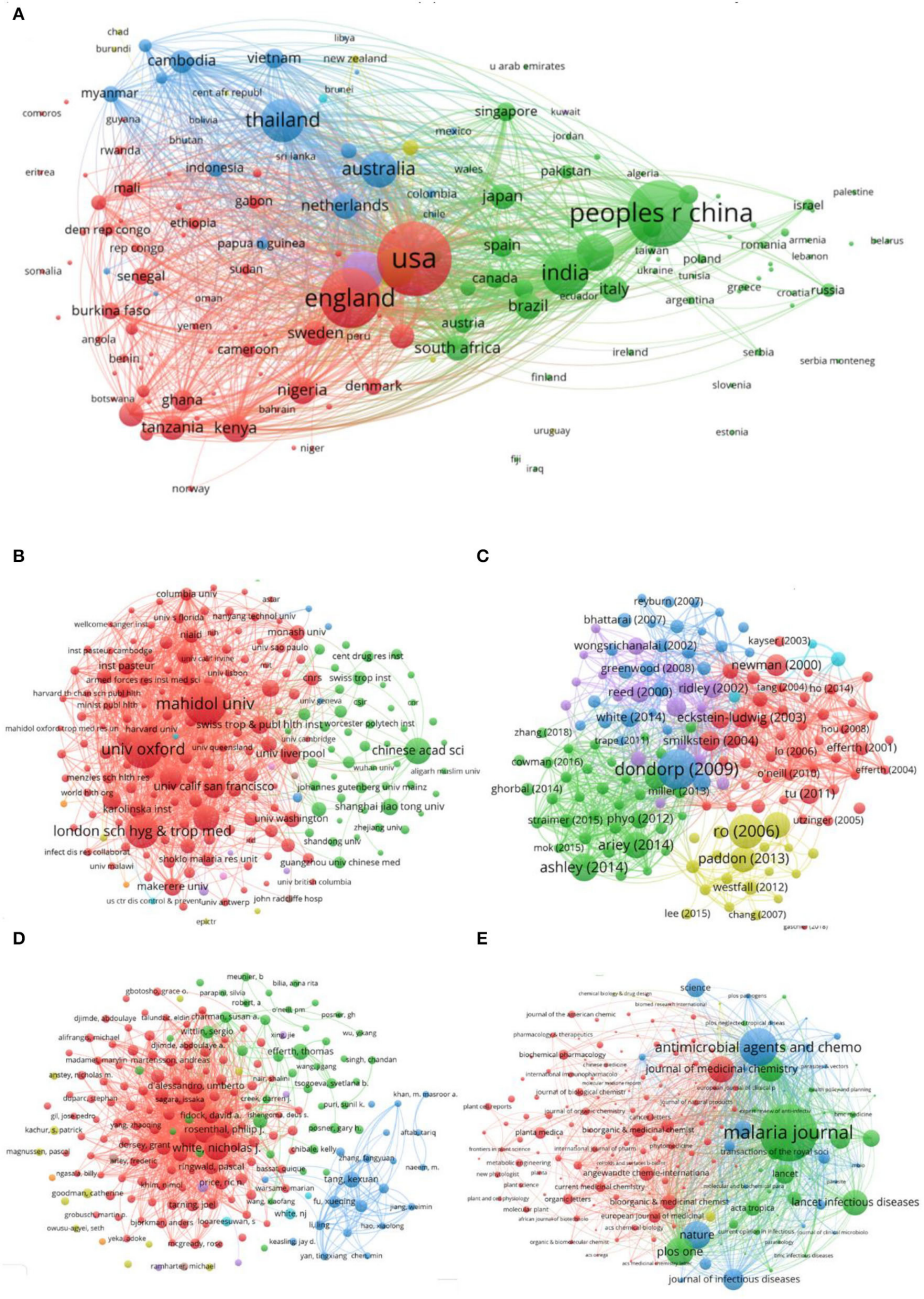
Bibliographic coupling analysis. **(A)** Network of co-occurrence of countries. **(B)** Network of co-occurrence of affiliations. **(C)** Network of co-occurrence of documents. **(D)** Network of co-occurrence of authors. **(E)** Network of co-occurrence of journals.

Bibliographic coupling denotes that if two documents cite the same references, they have a coupling relationship. In addition, on the basis of the latest version of Journal Citation Reports, the impact factor (IF), which has been widely used as the main indicator to measure the quality and impact of journals, was obtained (32). The network of bibliographic coupling analysis is shown in Figure 8, and the top 10 countries are shown in Supplementary Table 4. The United States had the most documents, citations, and total link strength. China ranked second in documents, but its total link strength was lower than that of England and Thailand. The citations of China were also lower than those in England. Except for the United States, China, and England, the documents of other countries were <1,000. In terms of affiliation (Supplementary Table 5), Mahidol University ranked first in terms of documents, citations, and total link strength, followed by the University of Oxford and London School of Hygiene & Tropical Medicine. Although the Chinese Academy of Sciences had high documents, its total link strength was lower than that of other institutions. As shown in Supplementary Table 6, White had the most documents, citations, and total link strength. Nosten ranked second in number of documents, but the author's total link strength was lower than that of Dondorp, Fidock, David and Menard; Dier had low documents, but their citations and total link strength were relatively high. The top 10 journals are shown in Supplementary Table 7. MALARIA JOURNAL has published the most documents, which have the most citations and total link strength, followed by ANTIMICROBIAL AGENTS and CHEMOTHERAPY and PLOS ONE. A paper written by Dondorp published in 2009 had the most citation, while the paper written by Ariey in 2014 had the highest total link strength (Supplementary Table 8).

### Analysis of Study on Artemisinin in the Treatment of Coronavirus Disease 2019

**Figure 9:**
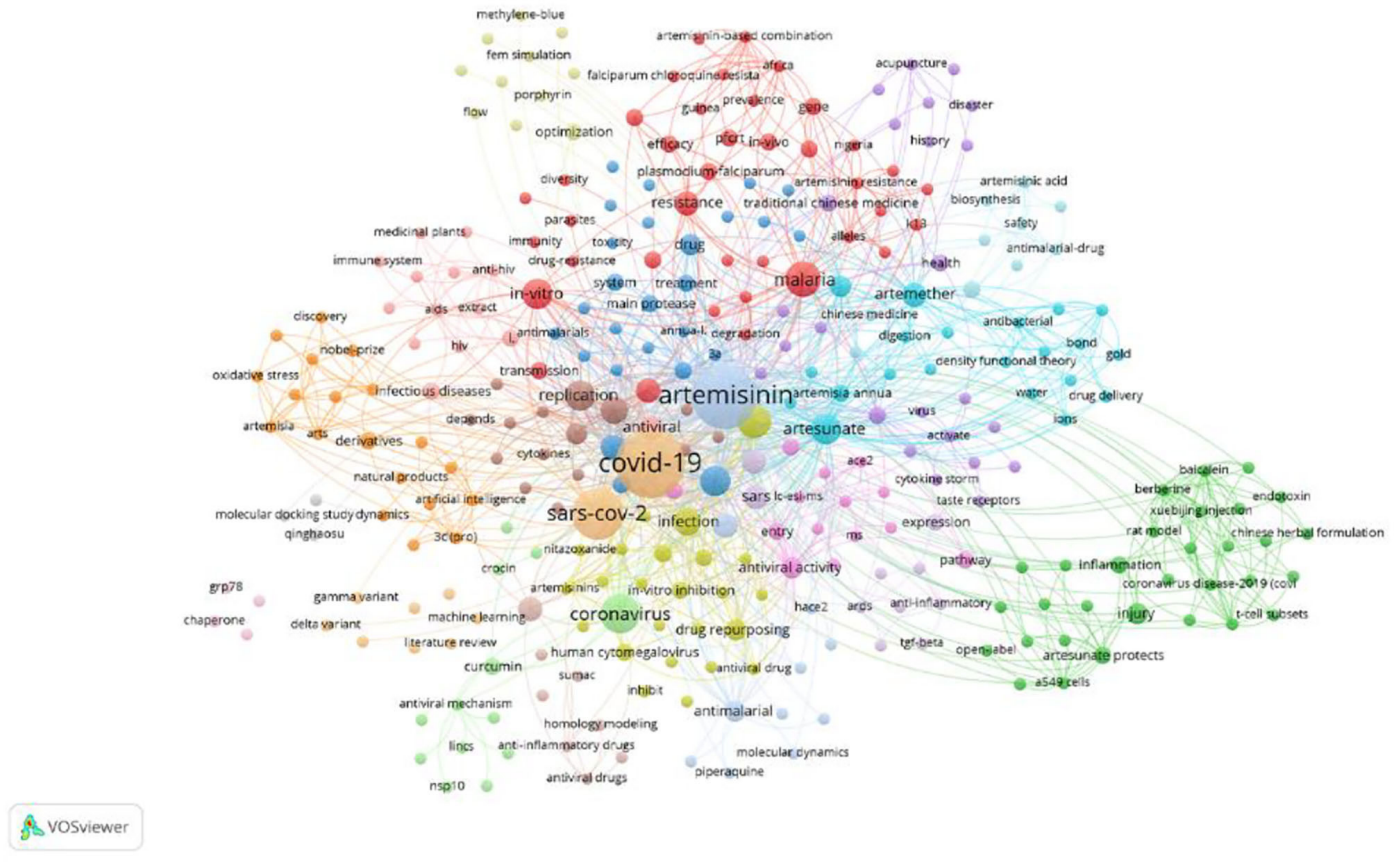
The network on keywords of artemisinin treating coronavirus disease 2019 (COVID-19).

During the coronavirus disease 2019 (COVID-19) pandemic, researchers are looking for effective treatments, and the therapeutic effect of artemisinin on COVID-19 was studied. A total of 48 papers about the therapeutic effect of artemisinin on COVID-19 were screened and analyzed in this study, with 316 keywords in total. As shown in Figure 9, except for COVID-19 and artemisinin, SARS-CoV-2, coronavirus, malaria, chloroquine, artesunate, hydroxychloroquine, and *in-vitro* appeared most frequently. Figure 9 shows the research on artemisinin for COVID-19 treatment, including the efficacy and mechanism of action *in vivo* and *in vitro*, immunity, gene expression, and cytokines.

## Discussion

Artemisinin, an antimalarial drug found in plants, is derived from Artemisia annua (2). Since 2001, artemisinin has been recommended by the WHO as the first-line drug for treating Falciparum malaria. However, the emergence of artemisinin resistance has attracted great attention in the academic community (33). In this study, bibliometric analysis was performed to explore the hotspots and developmental trends of studies on artemisinin on the basis of WoSCC with VOSviewer and Citespace. A total of 8,466 original articles and reviews published from 2000 to 2021 were retrieved in this study. The United States ranked first in terms of NP among all countries/regions, indicating that the country had a high production in the research of artemisinin. Although artemisinin was first discovered by Chinese scholars, China ranked second in terms of NP, indicating that the country needs to strengthen its research on artemisinin. The H-index, average per item, and NC of China were lower than those of English and Thailand. The average per item of Switzerland, France, Germany, and Australia was higher than that of China, indicating that the quality of papers in China needs to be improved.

Three Thai authors and three English affiliations came in the top 10 authors and affiliations in the research of artemisinin, respectively, indicating that the best affiliations were in England, and Thailand possessed the most professional researchers around the world. Compared with the United States, England and Thailand had a relatively high average per item. This finding showed that the research quality in England and Thailand was high.

Among the top 10 productive journals, only two had notably high IF, indicating that the quality of research papers in this field needs improvement and the depth of artemisinin research should be strengthened. MALARIA JOURNAL, which is a specialist journal related to malaria and has a certain influence in the field of malaria, published the greatest number of papers related to artemisinin. This finding showed that the research on artemisinin mainly focuses on antimalaria. However, the IF of this journal was not high (2.979), and the possible reason is that compared with NATURE, the LANCET, NEW ENGLAND JOURNAL OF MEDICINE, or other top journals, MALARIA JOURNAL has moderately low requirements for the amount of data and innovation of articles, and the publishing cycle is not too long. Considering the difficulty of publication, most scholars published articles in MALARIA JOURNAL, also indicating that the journal had a powerful impact in the field of artemisinin research.

Nine of the top 10 articles with the highest GCS were published in journals with higher IF, suggesting that these journals have published major breakthroughs in this field, reminding researchers interested in this field to pay close attention to these journals.

The signaling pathways involved in various mechanisms have been discovered with the deepening of research. The network of keywords showed that nuclear transcription factor (NF)-kB played an important role in the pharmacological mechanism of artemisinin. Analysis of keywords indicated that in recent years, the research on artemisinin biosynthesis has become popular. The main reason is that artemisinin was widely used, and clinical demand was large. Artemisia annua was the only plant source of artemisinin (34), but the content of artemisinin is very low. Thus, developing new methods to produce artemisinin to meet the increasing demand is necessary (35), and finding new methods to obtain artemisinin has become a research hotspot. Artemisinin is a kind of sesquiterpene, which is synthesized by the isoprenoid pathway, and farnesyl pyrophosphate (FPP) is its main synthetic precursor. The synthesis of amorphadiene is catalyzed by FPP amorphadiene synthase. Then, amorphadiene is oxidized by CYP450 enzyme to produce artemisinic acid (35). Artemisinin is produced from artemisinic acid under the action of photooxidation (36).

The total synthesis of artemisinin began in the 1980's. More than one synthesis and purification method of artemisinin was reported (37–40). Paddon designed a method to synthesize artemisinin from artemisinic acid. This method was economical and efficient. The yield of artemisinin was 40–50%, and the purity was higher than that of artemisinin extracted from plants. This method has important application prospects and market value (25).

In 2006, WHO recommended ACTs as the first-line drug for the treatment of Falciparum malaria. Due to its obvious curative effect, artemisinin could effectively reduce the infection rate and mortality rate of malaria and control its prevalence (41). Peroxy is the pharmacophore of artemisinin for treating malaria. At present, the mechanisms of artemisinin for treating malaria mainly include the carbon-free radical hypothesis, heme target hypothesis, calcium pump hypothesis, mitochondrial target hypothesis, and heme-activated multi-target hypothesis (42).

Further research showed that artemisinin not only has antimalaria pharmacological effects but also antitumor, antifibrosis, anti-inflammatory, and bactericidal effects. The works of Hou et al. showed that artesunate remarkably improved autoimmune arthritis by selectively reducing germinal center B cells (43). Kalvin et al. demonstrated that artemisinin could inhibit proliferation of endometrial cancer and downregulation of CDK4 expression by interference interaction of CDK4 promoter with NF-κB interactions (44). In addition, many studies have confirmed that artemisinin had inhibitory effects on lung cancer, liver cancer, cervical cancer, and other malignant tumors (45–51). Artemisinin could also abrogate dextran sulfate sodium-induced intestinal inflammation, and by inducing CYP3A expression *via* activation of pregname X receptor, artemisinin prevented or reduced the severity of colonic inflammation (52). Wang investigated the antiviral activity of artemisinin by measuring the hallmark features of viral replication *in vitro* and *in vivo*. Enhancing host type I interferon response was associated with the antiviral effect of artemisinin (53). Artemisinin also has inhibitory effects on *Alternaria tabacum, Escherichia coli, Staphylococcus aureus*, and other bacteria and fungi, but most of the studies were performed *in vitro* (54–56). The antibacterial research of artemisinin is still in its infancy. The next research should focus on *in vivo* research and action mechanism.

Comparison between Figures 7A,B revealed that the mechanism of artemisinin resistance has become a hotspot over the last years. The definition of artemisinin resistance is as follows: after artesunate monotherapy or ACT treatment of patients with malaria, the elimination half-life of *Plasmodium* in patients' blood is ≥5h (57). The first artemisinin-resistant case was found at the Thai-Cambodian border from 2003 to 2004. In 2008, another case of resistance of *Plasmodium* to artemisinin was found in Beilin Province, Cambodia (58). The APY of “Western Cambodia” and “Cambodia” was 2,016, which indicated that several studies on artemisinin resistance in Cambodia began in 2016. The gene mutations of *Plasmodium*, such as the multidrug resistance gene (pfmdr1) and chloroquine resistance transmembrane transporter gene (pfcrt), could affect its sensitivity to artemisinin, which is closely associated with the emergence of drug resistance. The keywords “pfmdr1” and “pfcrt” were included in cluster 1, which was mainly about artemisinin resistance, and the APY of the two keywords was 2,015. At present, the main criteria for evaluating artemisinin resistance are as follows: treatment failure and delayed parasite clearance on the third day (59). Whether artemisinin resistance could worsen in the future needs further observation. Birnbaum's results showed that the mechanism of drug resistance was related to the mutation of K13 and the decreased concentration of hemoglobin degradation products (60). In addition, a response mechanism of unfolded protein was found in *Plasmodium falciparum* (61). The appearance and endemic transmission of artemisinin-resistant *Plasmodium falciparum* were identified in Africa. However, K13 was not the best predictor for artemisinin resistance in Africa (62–64), suggesting that the artemisinin-resistant *Plasmodium falciparum* in Africa may be different from that in Cambodia. Thus, the mechanism and molecular markers of artemisinin resistance in Africa need further research. To prevent its spread, first, improving the local medical and health conditions and enhancing people's awareness of antimalaria are necessary. Second, researchers should do a good job in the research and innovation of artemisinin, study the joint application between artemisinin and other drugs, and strengthen the research and development of diversified and effective artemisinin compound drugs to keep *Plasmodium* highly sensitive to artemisinin and prevent the spread of artemisinin resistance. Finally, antimalarial drugs should be used reasonably in the clinic. The use of unnecessary antimalarial drugs must be limited, and new antimalarial drugs with no toxic side effects must be actively developed.

COVID-19 has spread to more than 210 countries and regions, bringing serious adverse effects to human survival and social development. Researchers and medical workers are also actively looking for effective treatments. On the basis of the anti-inflammatory, immunomodulatory, and antifibrosis effects of artemisinin, researchers have proposed to exploit its potential medicinal value for the treatment of COVID-19 (65). The results of Mathieu Gendrot et al. showed that for the treatment of COVID-19, mefloquine-artesunate exerted the highest antiviral activity. Artemether-lumefantrine, artesunate-amodiaquine, dihydroartemisinin-piperaquine, or artesunate-pyronaridine also showed antiviral effect (66). “Baicalein,” “berberine,” “curcumin,” “emodin,” “myricetin,” and “ginsenosides” appeared in Figure 9, indicating that artemisinin may be combined with these compounds to treat COVID-19.

In addition, Figure 9 shows that the pharmacological effects of artemisinin against COVID-19 included anti-inflammatory, antioxidant, immune regulation, regulation of autophagy, antivirus, and protection of myocardium, suggesting that artemisinin may have certain effects on improving immunity, reducing the degree of pulmonary fibrosis and pulmonary inflammation, and alleviating injury of organs and tissues of patients infected with COVID-19. However, at present, the research on the clinical safety and effectiveness of artemisinin in the treatment of COVID-19 is still blank, and the research and verification of the inhibitory effect of artemisinin on the SARS-CoV-2 virus and the effect of reducing the degree of pulmonary fibrosis and effectively alleviating the inflammatory response of patients should be strengthened. By means of performing in-depth research, artemisinin could provide a reference for exploring effective drugs for treating COVID-19, and the clinical application of artemisinin could be expanded. Bibliometric analysis and visualization of hotspots and development trends could not only reveal further information but also provide valuable explorations in the field of artemisinin research. However, this study has some limitations. First, only English reviews and articles from WoSCC were included. Second, failing to analyze the full text of a publication, VOSviewer may miss some information. Finally, due to the exclusion of some new publications, this study may have a lag to some extent.

## Conclusion

In this study, the most relevant authors, the most cited published papers, authors' research outputs, leading journals, and relevant countries in the publications of artemisinin from 2000 to 2021 were analyzed quantitatively and qualitatively for visualization and evaluation of the findings on artemisinin research. Over the past 22 years, the NPs on artemisinin steadily increased. The United States and China had a major impact on this field. Effective collaboration among different countries/regions could help promote artemisinin research. Hotspots associated with artemisinin included artemisinin resistance, molecular markers, polymorphisms, and drug discovery. The bibliometric analysis could effectively help researchers identify hotspots, frontiers, and new directions correlated with artemisinin research.

## Data Availability Statement

The raw data supporting the conclusions of this article will be made available by the authors, without undue reservation.

## Author Contributions

YD, LiL, JH, LZ, YW, JL, YL, and HL participated in the design of the study and wrote the manuscript. KZ and LuL collected and analyzed the data. XS, XW, and MZ edited the manuscript. XH and BZ consulted the relevant literature. All authors read and approved the final manuscript.

## Conflict of Interest

The authors declare that the research was conducted in the absence of any commercial or financial relationships that could be construed as a potential conflict of interest.

## Publisher's Note

All claims expressed in this article are solely those of the authors and do not necessarily represent those of their affiliated organizations, or those of the publisher, the editors and the reviewers. Any product that may be evaluated in this article, or claim that may be made by its manufacturer, is not guaranteed or endorsed by the publisher.

## References

[1] Liao Y Wang Y Luo X Luo K Wan S Qing C. Research progress on activation mechanism of artemisinin compounds in antitumor effects. Chin Tradit Herb Drugs. (2021) 52:3429–35.

[2] Tu Y. The discovery of artemisinin (qinghaosu) and gifts from Chinese medicine. Nat Med. (2011) 17:1217–20. 10.1038/nm.2471 21989013

[3] Banek K Lalani M Staedke SG Chandramohan D. Adherence to artemisinin-based combination therapy for the treatment of malaria: a systematic review of the evidence. Malar J. (2014) 13:1–13. 10.1186/1475-2875-13-7 24386988 PMC3893456

[4] Wang J Xu C Wong YK Li Y Liao F Jiang T. Artemisinin, the magic drug discovered from traditional Chinese medicine. Engineering. (2019) 5:32–9. 10.1016/j.eng.2018.11.011

[5] Lai H Sasaki T Singh NP. Targeted treatment of cancer with artemisinin and artemisinin-tagged iron-carrying compounds. Expert Opin Ther Targets. (2005) 9:995–1007. 10.1517/14728222.9.5.995 16185154

[6] Zhong CL Yao ZJ. Synthesis of the biotinylated artemisinin derivative. Acta Chimica Sinica. (2008) 66:1074–8. 26340163

[7] Sriram D Devakaram RV Dinakaran M Yogeeswari P. Aromatic amino analogues of artemisinin: synthesis and *in vivo* antimalarial activity. Med Chem Res. (2010) 19:524–32. 10.1007/s00044-009-9209-5

[8] Liu YG Lok CN Ko BCB Shum TYT Wong MK Che CM. Subcellular Localization of a fluorescent artemisinin derivative to endoplasmic reticulum. Org Lett. (2010) 12:1420–3. 10.1021/ol902890j 20192248

[9] Wang Y Li Y Shang D Efferth T. Interactions between artemisinin derivatives and P-glycoprotein. Phytomedicine. (2019) 60:152998. 10.1016/j.phymed.2019.152998 31301971

[10] Haldar K Bhattacharjee S Safeukui I. Drug resistance in plasmodium. Nat Rev Microbiol. (2018) 16:156–70. 10.1038/nrmicro.2017.161 29355852 PMC6371404

[11] Zhai K Ma W Huang T. Hot spots and trends in knee revision research since the 21st century: a bibliometric analysis. Ann Transl Med. (2021) 9:388. 10.21037/atm-20-3969 33842609 PMC8033385

[12] Demir E Yasar E Ozkocak V Yildirim E. The evolution of the field of legal medicine: a holistic investigation of global outputs with bibliometric analysis. J Forensic Leg Med. (2020) 69:1–9. 10.1016/j.jflm.2019.101885 31733463

[13] Wang S Zhou H Zheng L Zhu W Zhu L Feng D. Global trends in research of macrophages associated with acute lung injury over past 10 years: a bibliometric analysis. Front Immunol. (2021) 12:669539. 10.3389/fimmu.2021.669539 34093568 PMC8173163

[14] Ding Y Chowdhury GG Foo S. Bibliometric cartography of information retrieval research by using co-word analysis. Inf Process Manag. (2001) 37:817–42. 10.1016/S0306-4573(00)00051-0

[15] Zhou Q Pei J Poon J Lau AY Zhang L Wang Y. Worldwide research trends on aristolochic acids (1957-2017): Suggestions for researchers. PLos ONE. (2019) 4:e0216135. 10.1371/journal.pone.0216135 31048858 PMC6497264

[16] Chen P Lin X Chen B Zheng K Lin C Yu B. The global state of research and trends in osteomyelitis from 2010 to 2019: a 10-year bibliometric analysis. Ann Palliat Med. (2021) 10:3726–38. 10.21037/apm-20-1978 33832287

[17] Igwaran A Edoamodu CE. Bibliometric analysis on tuberculosis and tuberculosis-related research trends in Africa: a decade-long study. Antibiotics. (2021) 10:423. 10.3390/antibiotics10040423 33921235 PMC8069363

[18] Wang Y Zhao N Zhang X Li Z Liang Z Yang J. Bibliometrics analysis of butyrophilins as immune regulators 1992-2019 and implications for cancer prognosis. Front Immunol. (2020) 11:1187. 10.3389/fimmu.2020.01187 32695099 PMC7338374

[19] Hirsch JE. An index to quantify an individual's scientific research output. Proc Natl Acad Sci U S A. (2005) 102:16569–72. 10.1073/pnas.0507655102 16275915 PMC1283832

[20] Bar-Ilan J. Rankings of information and library science journals by JIF and by h-type indices. J Informetr. (2010) 4:141–7. 10.1016/j.joi.2009.11.006

[21] Liu T Yang L Mao H Ma F Wang Y Zhan Y. Knowledge domain and emerging trends in podocyte injury research from 1994 to 2021: a bibliometric and visualized analysis. Front Pharmacol. (2021) 12:772386. 10.3389/fphar.2021.772386 34925030 PMC8678497

[22] Wei Q Shen J Wang D Han X Shi J Zhao L. A bibliometric analysis of researches on flap endonuclease 1 from 2005 to 2019. BMC Cancer. (2021) 21:374. 10.1186/s12885-021-08101-2 33827468 PMC8028219

[23] Ashley EA Dhorda M Fairhurst RM Amaratunga C Lim P Suon S. Spread of Artemisinin Resistance in Plasmodium falciparum Malaria. N Engl J Med. (2014) 371:411–23. 10.1056/NEJMoa1314981 25075834 PMC4143591

[24] Ariey F Witkowski B Amaratunga C Beghain J Langlois AC Khim N. A molecular marker of artemisinin-resistant Plasmodium falciparum malaria. Nature. (2014) 505:50–5. 10.1038/nature12876 24352242 PMC5007947

[25] Paddon CJ Westfall PJ Pitera DJ Benjamin K Fisher K McPhee D. High-level semi-synthetic production of the potent antimalarial artemisinin. Nature. (2013) 496:528–3. 10.1038/nature12051 23575629

[26] White NJ Pukrittayakamee S Tran Tinh H Faiz MA Mokuolu OA Dondorp AM. Malaria. Lancet. (2014) 383:723–35. 10.1016/S0140-6736(13)60024-0 23953767

[27] Phyo AP Nkhoma S Stepniewska K Ashley EA Nair S McGready R. Emergence of artemisinin-resistant malaria on the western border of Thailand: a longitudinal study. Lancet. (2012) 379:1960–6. 10.1016/S0140-6736(12)60484-X 22484134 PMC3525980

[28] Bousema T Drakeley C. Epidemiology and infectivity of plasmodium falciparum and plasmodium vivax gametocytes in relation to malaria control and elimination. Clin Microbiol Rev. (2011) 24:377–410. 10.1128/CMR.00051-10 21482730 PMC3122489

[29] Westfall PJ Pitera DJ Lenihan JR Eng D Woolard FX Regentin R. Production of amorphadiene in yeast, and its conversion to dihydroartemisinic acid, precursor to the antimalarial agent artemisinin. Proc Natl Acad Sci U S A. (2012) 109:111–8. 10.1073/pnas.1110740109 22247290 PMC3271868

[30] Straimer J Gnaedig NF Witkowski B Amaratunga C Duru V Ramadani AP. K13-propeller mutations confer artemisinin resistance in plasmodium falciparum clinical isolates. Science. (2015) 347:428–31. 10.1126/science.1260867 25502314 PMC4349400

[31] Achan J Talisuna AO Erhart A Yeka A Tibenderana JK Baliraine FN. Quinine, an old anti-malarial drug in a modern world: role in the treatment of malaria. Malaria Journal 10 (2011). 10.1186/1475-2875-10-144 21609473 PMC3121651

[32] Achan J Talisuna AO Erhart A Yeka A Tibenderana JK Baliraine FN. Current concepts on bibliometrics: a brief review about impact factor, eigenfactor score, citescore, SCImago journal rank, source-normalised impact per paper, H-index, and alternative metrics. Ir J Med Sci. (2019) 188:939–51. 10.1007/s11845-018-1936-5 30511320

[33] Li S Li C-H Jiang T-L. Reasearch on lipid metabolism of Plasmodium and antimalarial mechanism of artemisinin. Zhongguo Zhong Yao Za Zhi. (2021) 46:4849–64. 10.19540/j.cnki.cjcmm.20210610.703 34581097

[34] Brown GD. The biosynthesis of artemisinin (Qinghaosu) and the phytochemistry of artemisia annua L. (Qinghao). Molecules. (2010) 15:7603–98. 10.3390/molecules15117603 21030913 PMC6259225

[35] Jiang F Gong T Chen J Chen T Yang J Zhu P. Synthetic biology of plants-derived medicinal natural products. Sheng Wu Gong Cheng Xue Bao. (2021) 37:1931–51. 10.13345/j.cjb.210138 34227286

[36] Tang K Shen Q Yan T Fu X. Transgenic approach to increase artemisinin content in Artemisia annua L. Plant Cell Rep. (2014) 33:605–15. 10.1007/s00299-014-1566-y 24413765

[37] Constantino MG Beltrame M daSilva GVJ. A novel asymmetric total synthesis of (+)-artemisinin. Synth Commun. (1996) 26:321–9. 10.1080/00397919608003621

[38] Yadav JS Babu RS Sabitha G. Stereoselective total synthesis of (+)-artemisinin. Tetrahedron Lett. (2003) 44:387–9. 10.1016/S0040-4039(02)02500-5

[39] Vil' VA Yaremenko IA Ilovaisky AI Terent'ev AO. Synthetic strategies for peroxide ring construction in artemisinin. Molecules. (2017) 22:117. 10.3390/molecules22010117 28085073 PMC6155923

[40] Piletska EV Karim K Cutler M Piletsky SA. Development of the protocol for purification of artemisinin based on combination of commercial and computationally designed adsorbents. J Sep Sci. (2013) 36:400–6. 10.1002/jssc.201200520 23203850

[41] Ashley EA Phyo AP. Drugs in development for malaria. Drugs. (2018) 78:861–79. 10.1007/s40265-018-0911-9 29802605 PMC6013505

[42] Luo W Liu Y Cong L Sun L Guo C. The research progress in artemisinin and its derivatives. Chinese J Med Chem. (2012) 22:155–66.

[43] Hou LF Huang HC. Immune suppressive properties of artemisinin family drugs. Pharmacol Ther. (2016) 166:123–7. 10.1016/j.pharmthera.2016.07.002 27411673 PMC5035609

[44] Tran KQ Tin AS Firestone GL. Artemisinin triggers a G1 cell cycle arrest of human Ishikawa endometrial cancer cells and inhibits cyclin-dependent kinase-4 promoter activity and expression by disrupting nuclear factor-kappa B transcriptional signaling. Anticancer Drugs. (2014) 25:270–81. 10.1097/CAD.0000000000000054 24296733 PMC4172338

[45] Vu Tuan K Le Huy B Phan Hai P Doan Thi H Nguyen Thi Thuy M Nguyen Hai N. Novel artemisinin-derived dimers: synthesis and evaluation of anticancer activities. Lett Drug Des Discov. (2017) 14:102–11. 10.2174/1570180813666160810155354

[46] Deng XR Liu ZX Liu F Pan L Yu HP Jiang JP. Holotransferrin enhances selective anticancer activity of artemisinin against human hepatocellular carcinoma cells. J Huazhong Univ Sci Technolog Med Sci. (2013) 33:862–5. 10.1007/s11596-013-1212-x 24337849

[47] Buragohain P Saikia B Surineni N Barua NC Saxena AK Suri N. Synthesis of a novel series of artemisinin dimers with potent anticancer activity involving Sonogashira cross-coupling reaction. Bioorg Med Chem Lett. (2014) 24:237–9. 10.1016/j.bmcl.2013.11.032 24332623

[48] Li X Gu S Sun D Dai H Chen H Zhang Z. The selectivity of artemisinin-based drugs on human lung normal and cancer cells. Environ Toxicol Pharmacol. (2018) 57:86–94. 10.1016/j.etap.2017.12.004 29227908

[49] Zhang Q Yi H Yao H Lu L He G Wu M. Artemisinin derivatives inhibit non-small cell lung cancer cells through induction of ROS-dependent apoptosis/ferroptosis. J Cancer. (2021) 12:4075–85. 10.7150/jca.57054 34093811 PMC8176242

[50] Gong XM Zhang Q Torossian A Cao JP Fu S. Selective radiosensitization of human cervical cancer cells and normal cells by artemisinin through the abrogation of radiation-induced G2 block. Int J Gynecol Cancer. (2012) 22:718–24. 10.1097/IGC.0b013e31824a67c9 22552829

[51] Li Y Zhou X Liu J Yuan X He Q. Therapeutic potentia and mechanisms of artemisinin and its derivatives for tumorigenesis and matestasis. Anticancer Agents Med Chem. (2020) 20:520–35. 10.2174/1871520620666200120100252 31958040

[52] Hu D Wang Y Chen Z Ma Z You Q Zhang X. Artemisinin protects against dextran sulfate-sodium-induced inflammatory bowel disease, which is associated with activation of the pregnane X receptor. Eur J Pharmacol. (2014) 738:273–84. 10.1016/j.ejphar.2014.04.050 24886881

[53] Wang X Zheng B Ashraf U Zhang H Cao C Li Q. Artemisinin inhibits the replication of flaviviruses by promoting the type I interferon production. Antiviral Res. (2020) 179:104810. 10.1016/j.antiviral.2020.104810 32360948

[54] Cui H Chen X Bai M Han D Lin L Dong M. Multipathway antibacterial mechanism of a nanoparticle-supported artemisinin promoted by nitrogen plasma treatment. ACS Appl Mater Interfaces. (2019) 11:47299–310. 10.1021/acsami.9b15124 31797661

[55] Huang M Shen J-Y Du C-C Yin T Ge F-J Tan Y-Q. Preliminary study on antibacterial activity of artemisinin and its derivatives. Zhongguo Zhong Yao Za Zhi. (2019) 44:1946–52. 10.19540/j.cnki.cjcmm.20190131.001 31342725

[56] Xu J Deng L Hu G Yang C Yu J Yang J. Study on antibacterial mechanism of artemisinin derivatives. Nat Prod Res Development. (2018) 30:725–30.

[57] Wongsrichanalai C. Artemisinin resistance or artemisinin-based combination therapy resistance? Lancet Infect Dis. (2013) 13:114–5. 10.1016/S1473-3099(12)70349-3 23347632

[58] Noedl H Se Y Schaecher K Smith BL Socheat D Fukuda MM. Evidence of artemisinin-resistant malaria in western cambodia. N Engl J Med. (2008) 359:2619–20. 10.1056/NEJMc0805011 19064625

[59] Chhibber-Goel J Sharma A. Profiles of Kelch mutations in Plasmodium falciparum across South Asia and their implications for tracking drug resistance. Int J Parasitol Drugs Drug Resist. (2019) 11:49–58. 10.1016/j.ijpddr.2019.10.001 31606696 PMC6796718

[60] Birnbaum J Scharf S Schmidt S Jonscher E Hoeijmakers WAM Flemming S et al A Kelch13-defined endocytosis pathway mediates artemisinin resistance in malaria parasites. Science. (2020) 367:51–9. 10.1126/science.aax4735 31896710

[61] Mok S Ashley EA Ferreira PE Zhu L Lin Z Yeo T. Population transcriptomics of human malaria parasites reveals the mechanism of artemisinin resistance. Science. (2015) 347:431–5. 10.1126/science.1260403 25502316 PMC5642863

[62] Kamau E Campino S Amenga-Etego L Drury E Ishengoma D Johnson K. K13-propeller polymorphisms in plasmodium falciparum parasites from Sub-Saharan Africa. J Infect Dis. (2015) 211:1352–5. 10.1093/infdis/jiu608 25367300 PMC4827505

[63] Djaman JA Olefongo D Ako AB Roman J Ngane VF Basco LK. Molecular epidemiology of malaria in cameroon and cote d'ivoire. xxxi. kelch 13 propeller sequences in plasmodium falciparum isolates before and after implementation of artemisinin-based combination therapy. Am J Trop Med Hyg. (2017) 97:222–4. 10.4269/ajtmh.16-0889 28719312 PMC5508902

[64] Madamet M Kounta MB Wade KA Lo G Diawara S Fall M. Absence of association between polymorphisms in the K13 gene and the presence of Plasmodium falciparum parasites at day 3 after treatment with artemisinin derivatives in Senegal. Int J Antimicrob Agents. (2017) 49:754–6. 10.1016/j.ijantimicag.2017.01.032 28450175

[65] Yang Y-M Chen L-N Qu S-Q Deng S-Q Liu H Wang X. Potential therapies for COVID-19 cardiovascular complications using artemisinin and its derivatives intervene based on its cardiovascular protection. Zhongguo Zhong Yao Za Zhi. (2020) 45:6053–64. 10.19540/j.cnki.cjcmm.20200828.601 33496147

[66] Gendrot M Duflot I Boxberger M Delandre O Jardot P Le Bideau M. Antimalarial artemisinin-based combination therapies (ACT) and COVID-19 in Africa: *In vitro* inhibition of SARS-CoV-2 replication by mefloquine-artesunate. Int J Infect Dis. (2020) 99:437–40. 10.1016/j.ijid.2020.08.032 32805422 PMC7426697

