# Supplementary material for: Worldwide Research Trends on Artemisinin: A Bibliometric Analysis From 2000 to 2021
Source: Front Med (Lausanne). 2022 May 6;9:868087. doi: 10.3389/fmed.2022.868087 (PMC9121127; doi:10.3389/fmed.2022.868087)
Supplement: Supplementary file 1 [file Data_Sheet_1.docx]

**Supplementary tables**

TABLE S1 The top 10 co-cited references in artemisinin research.

| Rank | Author | Citations | Total link strength | Source | Co-cited Reference | Year |
| --- | --- | --- | --- | --- | --- | --- |
| 1 | [Dondorp, AM](https://www.webofscience.com/wos/author/record/41338) | 1360 | 11779 | NEW ENGL J MED | Artemisinin Resistance in Plasmodium falciparum Malaria | 2009 |
| 2 | [KLAYMAN, DL](https://www.webofscience.com/wos/alldb/general-summary?queryJson=%5B%7B%22rowField%22:%22AU%22,%22rowText%22:%22KLAYMAN,%20DL%22%7D%5D&eventMode=oneClickSearch) | 940 | 5010 | SCIENCE | [QINGHAOSU (ARTEMISININ) - AN ANTIMALARIAL DRUG FROM CHINA](https://www.webofscience.com/wos/alldb/full-record/MEDLINE:3887571) | 1985 |
| 3 | Ashley, EA | 793 | 7627 | NEW ENGL J MED | [Spread of Artemisinin Resistance in Plasmodium falciparum Malaria](https://www.webofscience.com/wos/woscc/full-record/WOS:000339556900006) | 2014 |
| 4 | Ariey, F | 786 | 8538 | NATURE | [A molecular marker of artemisinin-resistant Plasmodium falciparum malaria](https://www.webofscience.com/wos/woscc/full-record/WOS:000329163300021) | 2014 |
| 5 | Noedl, H | 741 | 7473 | NEW ENGL J MED | [Evidence of Artemisinin-Resistant Malaria in Western Cambodia](https://www.webofscience.com/wos/woscc/full-record/WOS:000261534200032) | 2008 |
| 6 | [TRAGER, W](https://www.webofscience.com/wos/alldb/general-summary?queryJson=%5B%7B%22rowField%22:%22AU%22,%22rowText%22:%22TRAGER,%20W%22%7D%5D&eventMode=oneClickSearch) | 462 | 2858 | SCIENCE | [HUMAN MALARIA PARASITES IN CONTINUOUS CULTURE](https://www.webofscience.com/wos/alldb/full-record/MEDLINE:781840) | 1976 |
| 7 | Eckstein-Ludwig U | 460 | 4506 | NATURE | Artemisinins target the SERCA of Plasmodium falciparum | 2003 |
| 8 | White, NJ | 434 | 3293 | SCIENCE | [Qinghaosu (Artemisinin): The price of success](https://www.webofscience.com/wos/woscc/full-record/WOS:000255026100031) | 2008 |
| 9 | Phyo, AP | 413 | 4537 | LANCET | [Emergence of artemisinin-resistant malaria on the western border of Thailand: a longitudinal study](https://www.webofscience.com/wos/woscc/full-record/WOS:000304757700030) | 2012 |
| 10 | [Meshnick, SR](https://www.webofscience.com/wos/alldb/general-summary?queryJson=%5B%7B%22rowField%22:%22AU%22,%22rowText%22:%22Meshnick,%20SR%22%7D%5D&eventMode=oneClickSearch) | 377 | 2794 | Microbiol Rev | [Artemisinin and the antimalarial endoperoxides: From herbal remedy to targeted chemotherapy](https://www.webofscience.com/wos/alldb/full-record/MEDLINE:8801435) | 1996 |

TABLE S2 The top 10 co-cited authors in artemisinin research.

| Rank | Author | Citations | Total link strength |
| --- | --- | --- | --- |
| 1 | White, NJ | 2924 | 45050 |
| 2 | World Health, Organization | 2233 | 26279 |
| 3 | Efferth, T | 2103 | 20723 |
| 4 | Dondorp, AM | 2096 | 29549 |
| 5 | Posner, GH | 1575 | 33037 |
| 6 | Haynes, RK | 1413 | 30363 |
| 7 | O'neill, PM | 1259 | 26139 |
| 8 | Noedl, H | 1233 | 19445 |
| 9 | Meshnick, SR | 1223 | 20048 |
| 10 | Ashley, EA | 1199 | 17990 |

TABLE S3 Top 20 keywords in terms of frequency in artemisinin research.

| id | Keyword | Frequency | Total link strength |
| --- | --- | --- | --- |
| 1 | artemisinin | 3210 | 12817 |
| 2 | malaria | 2457 | 12299 |
| 3 | artesunate | 1106 | 6069 |
| 4 | plasmodium-falciparum | 1042 | 5079 |
| 5 | in-vitro | 971 | 4871 |
| 6 | artemisinin resistance | 926 | 4589 |
| 7 | plasmodium falciparum | 849 | 5315 |
| 8 | resistance | 829 | 4562 |
| 9 | artemether-lumefantrine | 687 | 4254 |
| 10 | derivatives | 678 | 3218 |
| 11 | plasmodium-falciparum malaria | 638 | 3692 |
| 12 | dihydroartemisinin | 621 | 3243 |
| 13 | chloroquine | 614 | 3773 |
| 14 | efficacy | 602 | 3639 |
| 15 | children | 530 | 2777 |
| 16 | antimalarial | 490 | 2357 |
| 17 | artemether | 396 | 2155 |
| 18 | expression | 391 | 1525 |
| 19 | pharmacokinetics | 385 | 2121 |
| 20 | drug | 375 | 1797 |

TABLE S4 The top 10 bibliographic coupling countries in artemisinin research.

| Rank | Country | Documents | Citations | Total link strength |
| --- | --- | --- | --- | --- |
| 1 | The US | 2036 | 89878 | 8968737 |
| 2 | China | 1635 | 38916 | 3402949 |
| 3 | England | 1334 | 65057 | 6455885 |
| 4 | India | 795 | 15975 | 2632689 |
| 5 | Thailand | 678 | 35907 | 4314229 |
| 6 | Switzerland | 623 | 26794 | 3031394 |
| 7 | France | 585 | 20714 | 2793775 |
| 8 | Germany | 502 | 19304 | 1857806 |
| 9 | Australia | 488 | 18628 | 2661171 |
| 10 | Nigeria | 273 | 5582 | 936429 |

TABLE S5 The top 10 bibliographic coupling affiliations in artemisinin research.

| Rank | Affiliation | Documents | Citations | Total link strength |
| --- | --- | --- | --- | --- |
| 1 | Mahidol University | 481 | 28501 | 1149 |
| 2 | University of Oxford | 421 | 20612 | 1137 |
| 3 | London School of Hygiene & Tropical Medicine | 330 | 11433 | 718 |
| 4 | Chinese Academy of Sciences | 225 | 6903 | 87 |
| 5 | French Ministry of Social Affairs and Health | 217 | 6529 | 413 |
| 6 | University of California, San Francisco | 179 | 7058 | 376 |
| 7 | The University of Liverpool | 146 | 7402 | 200 |
| 8 | Chinese Center for Disease Control and Prevention | 130 | 4632 | 288 |
| 9 | Swiss Tropical & Public Health Institute | 124 | 4844 | 367 |
| 10 | Makerere University | 120 | 3635 | 271 |

TABLE S6 The top 10 bibliographic coupling authors in artemisinin research.

| Rank | Author | Documents | Citations | Total link strength |
| --- | --- | --- | --- | --- |
| 1 | White, Nicholas J. | 126 | 10372 | 470870 |
| 2 | Nosten, Francois | 91 | 7185 | 330256 |
| 3 | Dondorp, Arjen M. | 76 | 7612 | 379014 |
| 4 | Rosenthal, Philip J. | 74 | 3493 | 262971 |
| 5 | Efferth, Thomas | 64 | 3500 | 89847 |
| 6 | Tang, Kexuan | 64 | 1549 | 133319 |
| 7 | D'alessandro, Umberto | 60 | 1815 | 142091 |
| 8 | Fidock, David A. | 58 | 4560 | 345328 |
| 9 | Menard, Didier | 58 | 2979 | 312950 |
| 10 | Ringwald, Pascal | 54 | 4042 | 259727 |

TABLE S7 the Top 10 bibliographic coupling journals in artemisinin research.

| Rank | Source | Documents | Citations | Total link strength |
| --- | --- | --- | --- | --- |
| 1 | MALARIA JOURNAL | 1021 | 22235 | 1211596 |
| 2 | ANTIMICROBIAL AGENTS AND CHEMOTHERAPY | 337 | 11637 | 673000 |
| 3 | PLOS ONE | 231 | 7161 | 327891 |
| 4 | AMERICAN JOURNAL OF TROPICAL MEDICINE AND HYGIENE | 227 | 6223 | 366914 |
| 5 | JOURNAL OF MEDICINAL CHEMISTRY | 124 | 6388 | 203536 |
| 6 | BIOORGANIC & MEDICINAL CHEMISTRY LETTERS | 111 | 2586 | 134038 |
| 7 | SCIENTIFIC REPORTS | 88 | 1431 | 185559 |
| 8 | ACTA TROPICA | 86 | 2602 | 142380 |
| 9 | MOLECULES | 84 | 1985 | 110127 |
| 10 | BIOORGANIC & MEDICINAL CHEMISTRY | 81 | 2253 | 96216 |

TABLE S8 The top 10 bibliographic coupling documents in artemisinin research.

| Rank | Document | Author | Citations | Total link strength | Journal | Year |
| --- | --- | --- | --- | --- | --- | --- |
| 1 | Artemisinin Resistance in Plasmodium falciparum Malaria | Dondorp, AM | 2223 | 185 | NEW ENGLAND JOURNAL OF MEDICINE | 2009 |
| 2 | [Production of the antimalarial drug precursor artemisinic acid in engineered yeast](https://www.webofscience.com/wos/alldb/full-record/WOS:000236736700042) | [Ro, DK](https://www.webofscience.com/wos/alldb/general-summary?queryJson=%5B%7B%22rowField%22:%22AU%22,%22rowText%22:%22Ro,%20DK%22%7D%5D&eventMode=oneClickSearch) | 1721 | 73 | NATURE | 2006 |
| 3 | Spread of Artemisinin Resistance in Plasmodium falciparum Malaria | Ashley, EA | 1231 | 229 | NEW ENGLAND JOURNAL OF MEDICINE | 2014 |
| 4 | A molecular marker of artemisinin-resistant Plasmodium falciparum malaria | Ariey, F | 1130 | 270 | NATURE | 2014 |
| 5 | Engineering a mevalonate pathway in Escherichia coli for production of terpenoids | Martin, VJJ | 1110 | 47 | \| NATURE BIOTECHNOLOGY | 2003 |
| 6 | High-level semi-synthetic production of the potent antimalarial artemisinin | Paddon, CJ | 1081 | 65 | NATURE | 2013 |
| 7 | Spiroindolones, a Potent Compound Class for the Treatment of Malaria | Rottmann, M | 839 | 160 | SCIENCE | 2010 |
| 8 | The influence of natural products upon drug discovery | Newman, DJ | 827 | 33 | NATURAL PRODUCT REPORTS | 2000 |
| 9 | Artemisinins target the SERCA of Plasmodium falciparum | Eckstein-Ludwig, U | 754 | 110 | NATURE | 2003 |
| 10 | Simple and inexpensive fluorescence-based technique for high-throughput antimalarial drug screening | smilkstein | 717 | 10 | ANTIMICROBIAL AGENTS AND CHEMOTHERAPY | 2004 |
